# Pomegranate fruit juice adulteration with apple juice: detection by UV–visible spectroscopy combined with multivariate statistical analysis

**DOI:** 10.1038/s41598-022-07979-7

**Published:** 2022-03-25

**Authors:** Lucia Pappalardo

**Affiliations:** Department of Biology, Chemistry and Environmental Sciences, American University of Sharjah, PO Box 26666, Sharjah, United Arab Emirates

**Keywords:** Nutrition, Chemistry

## Abstract

Pomegranate is rich in high value nutritional substances known to be beneficial against several diseases and its use in medicine is known since ancient times. Due to its properties and delicious taste, pomegranate fresh fruit juices demand has been growing worldwide and its adulteration is becoming a problem. Low-cost, user friendly and fast detection methods are therefore desirable in order to easily and rapidly detect adulteration of short shelf-life fresh fruit juices. For this purpose fresh squeezed arils pomegranate juice samples adulterated with less expensive apple juice concentrate were investigated by UV–visible spectroscopy combined with multivariate statistical analysis. Unsupervised principle component analysis (PCA), supervised projection to latent structure discriminant analysis (PLS-DA) and orthogonal projection to latent structure discriminant analysis (OPLS-DA) were performed on the full spectra. OPLS-DA analysis of UV–visible spectra proved to be a suitable method to detect pomegranate juices adulterated by more than 20% v/v apple juice concentrate.

## Introduction

Pomegranate has been widely cultivated in the Mediterranean since ancient times and nowadays it is widely cultivated also in Middle East and Asia. Pomegranate fresh fruit juice demand is increasing due to its health benefits and it is very popular among consumers concerned about healthy diet and lifestyle. The oldest report about medical applications of pomegranate dates back to 1500 B.C. that mentioned its use in Ancient Egypt to treat several infections^1^. Recent studies have reported pomegranate’s antiviral, antimicrobial, antioxidative and anti-inflammatory properties and potential beneficial applications against several diseases^2^. As the demand of fresh fruit juices with high nutritional values is increasing worldwide so their adulteration is becoming a growing problem. Therefore, low-cost, user friendly and fast adulteration detection methods are desirable in order to control easily and rapidly the quality of the short shelf-life fresh fruit juices.

Studies based on mass spectrometry (MS) combined with multivariate statistical analysis for determining the level of adulteration in fruit juices^3–5^ and in particular in pomegranate fruit juices^6^ have been performed. ^1^H nuclear magnetic resonance (NMR) analyses combined with chemometrics have been applied to determine the percentage of pure fruit juices in blends of apple, orange, pineapple and pomegranate juices^7^ and adulterations in grape nectars^8^. A method based on Fourier transform infrared (FTIR) spectroscopy combined with unsupervised principal component analysis (PCA) was able to determine adulteration in pomegranate juices concentrate with grape juice concentrate^9^. Furthermore a comprehensive study by UV–Vis spectroscopy and PCA analysis on pomegranate juices mixed with grape and apple juices or simply diluted with water has being published^10^. Boggia et al*.* concluded that pomegranate adulterated with these fillers can be detected by UV–Vis spectroscopy and unsupervised PCA analysis. However, they determined that this method was not particularly distinctive for pomegranate and apple juices mixtures.

The aim of this work was to expand on the findings of Boggia et al*.* and investigate how well pomegranate juices adulterated by apple juices can be detected by applying supervised multivariate statistical discriminant analysis (PLS-DA and OPLS-DA) to UV–Vis spectra of fruit juices and their mixtures. For this purpose, fresh squeezed arils pomegranate juice samples adulterated with less expensive apple juice concentrate were investigated by UV–visible spectroscopy combined with supervised and unsupervised multivariate statistical analysis.

## Material and methods

### Samples

Fruit juices were purchased at local grocery stores. Fresh squeezed arils pomegranate juices (n = 56), and apple juices prepared from concentrate (n = 56) were used. The pomegranate and apple juices were purchased, respectively, from two different companies. For each of these juice samples, mixtures in their native fresh conditions at 10%, 20%, 30% and 40% v/v apple/pomegranate juices were prepared by mixing in a vial. Mixtures and pure juices were further diluted 20 times with Milli-Q water (Merck Millipore, Burlington, MA, USA) and 5 ml of each sample were centrifuged at 4000 rpm for 10 min prior to analysis. The dilution and centrifugation were required in order to be able to detect the UV–Vis signal in the proper range of the instrument.

### Instrumentation

Spectra were acquired on a Shimadzu UV-1800 spectrometer equipped with the software UVProbe ver. 2.43 (Shimadzu, Kyoto, Japan). The temperature of the samples was controlled by a TC1 temperature controller and T-App software ver. 1.30 (Quantum Northwest Inc., Liberty Lake, WA, USA).

### Data acquisition

UV–Vis absorption spectra were acquired in the 200–800 nm range with 1 nm resolution. Spectra for each sample were recorded at 10 different temperatures in the range 25–80 °C. Two spectra were recorded for each sample at each temperature and their average values were employed for further analysis. Quartz cuvettes of 1 cm path length were used. Spectra showed stability over the temperature range investigated and only data recorded at 25 °C are reported here.

### Multivariate statistical analysis

The UV–Vis spectra were exported to MS Excel software. The full spectra range from 200 to 800 nm was used for the analysis and the size of each spectrum was 601 data points. Unsupervised principle component analysis (PCA) was performed initially, followed by supervised projection to latent structure discriminant analysis (PLS-DA) and orthogonal projection to latent structure discriminant analysis (OPLS-DA) using the SIMCA software (v.13.5, Umetrics, Umeå, Sweden)^11^. OPLS-DA uses an orthogonal filter to remove all the information in X that are not correlated with Y and is able to augment the classification performance of PLS-DA^12^.

Univariate (UV) scaling was used for all of the variables after mean centering^13^. Pareto scaling and mean centering without scaling were also investigated on the data set under analysis and UV scaling provided the best results. Validation was accomplished using the routine in SIMCA taking 7th-order Monte Carlo cross-validation^11,14^. Goodness of fit of the data to the model [R^2^ (cum)] and predictability of the model [Q^2^ (cum)] were calculated for the scores plots and variables of importance determined by comparing coefficient plots which resemble the real spectral features^15^.

## Results and discussion

Pomegranate juices have high nutritional values and are sometimes adulterated by counterfeiters by the addition of less expensive juices as for example apple juices. While sophisticated techniques as MS^3–6^, NMR^7,8^, FTIR^9^ have proved to be effective in detecting the adulterations in fruit juices, it is desirable to have a faster and less expensive detection method as for example UV–Vis spectroscopy. For this reason, fresh squeezed pomegranate juices, apple juices prepared from concentrate and their mixtures, in their native conditions, were investigated by UV–Vis spectroscopy and full-resolution spectra were analyzed by multivariate statistics in this study.

**Figure 1:**
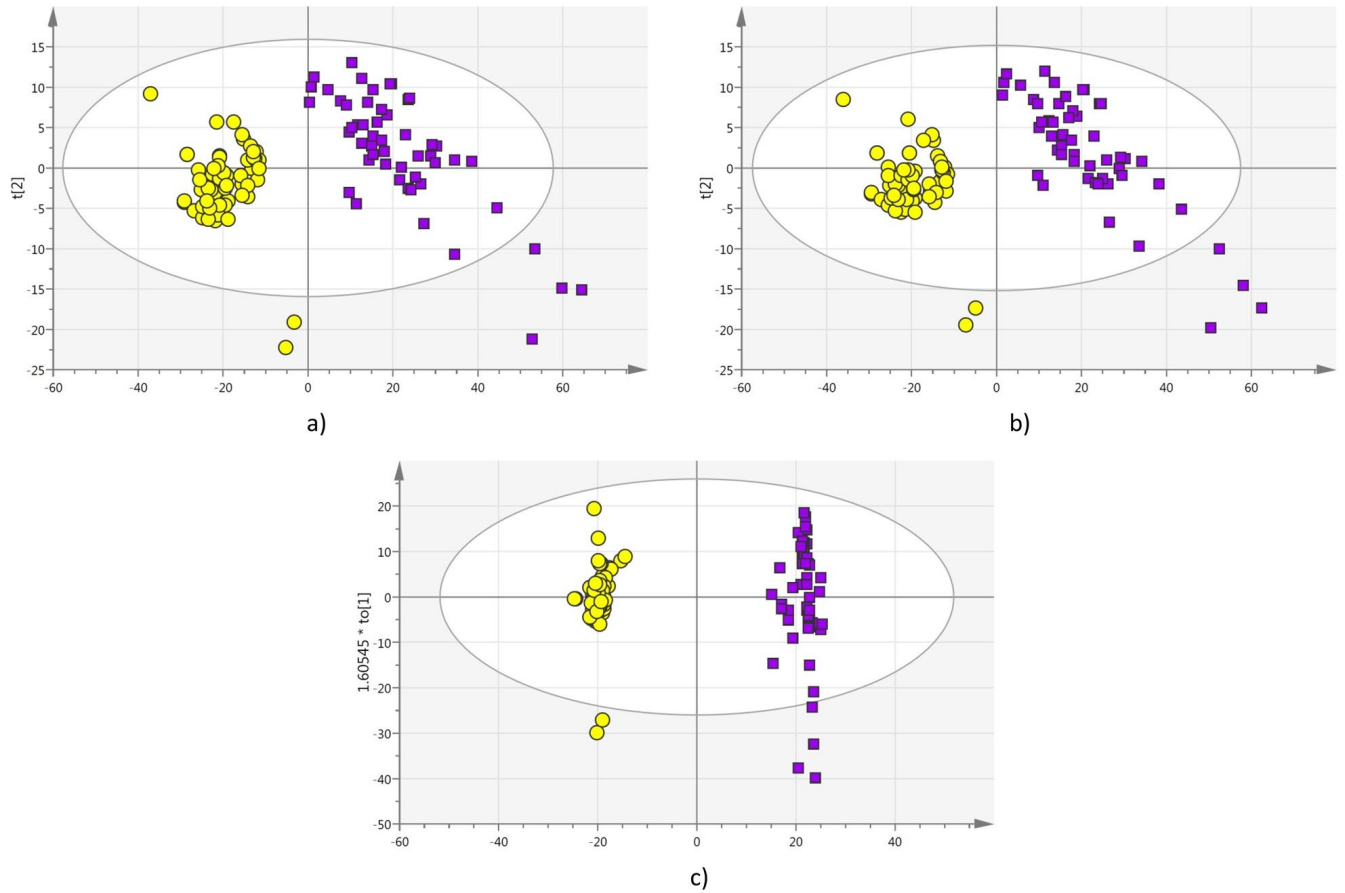
(**a**) PCA Scores plot; (**b**) PLS-DA Scores plot; (**c**) OPLS-DA Scores plot; analysis comparing UV–Vis spectra of apple juice samples (circles) to those of pomegranate juices (squares).

First goal was to investigate if the method was able to differentiate between apple and pomegranate juices. For this scope the UV–Vis spectra of 100% pomegranate and 100% apple juices samples were recorded and then analyzed by multivariate methods. Unsupervised PCA was calculated and the PCA scores plot showed good clustering of the two classes (Fig. 1a). Then the two classes, apple (A) and pomegranate (P), were specified for the discriminant analysis (PLS-DA and OPLS-DA). The corresponding PLS-DA (Fig. 1b) and OPLS-DA (Fig. 1c) scores plots show a clear separation, quite pronounced in the OPLS results. All observations fell within or close to the 95% Hotelling ellipse, which indicates a coherent dataset (Fig. 1). The analysis also showed excellent cluster correlation [R^2^ (cum) = 0.99] and predictability [Q^2^ (cum) = 0.99] with good validation results. This first set of experiments proved that UV-spectroscopy combined with multivariate statistical analysis was able to differentiate between apple and pomegranate juices.

**Figure 2:**
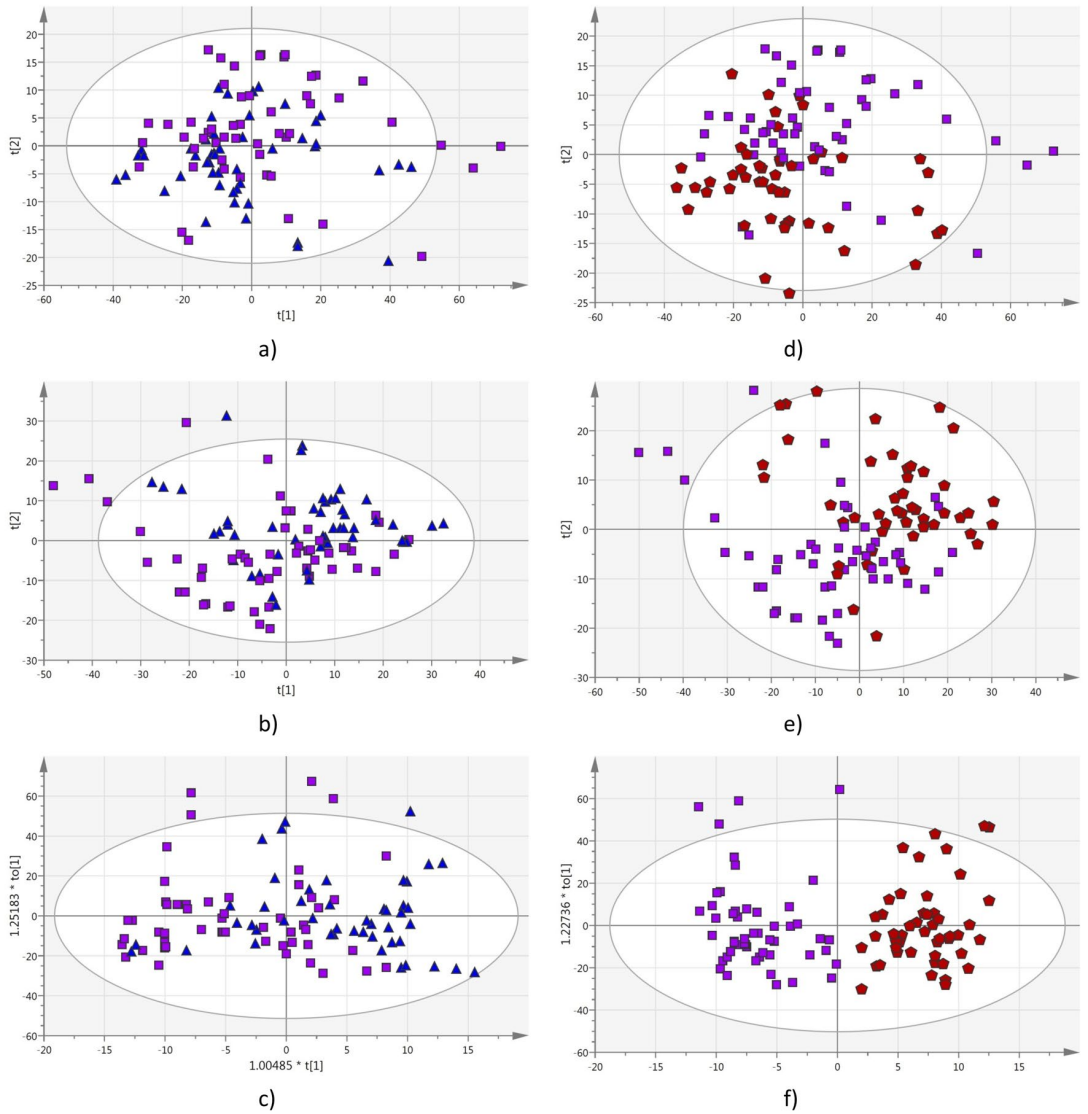
(**a**) PCA Scores plot; (**b**) PLS-DA Scores plot; (**c**) OPLS-DA Scores plot; analysis comparing UV–Vis spectra of pomegranate juices (squares) to those of 10% (triangles) mixtures. (**d**) PCA Scores plot; (**e**) PLS-DA Scores plot; (**f**) OPLS-DA Scores plot; analysis comparing UV–Vis spectra of pomegranate juices (squares) to those of 20% (pentagons) mixtures.

**Figure 3:**
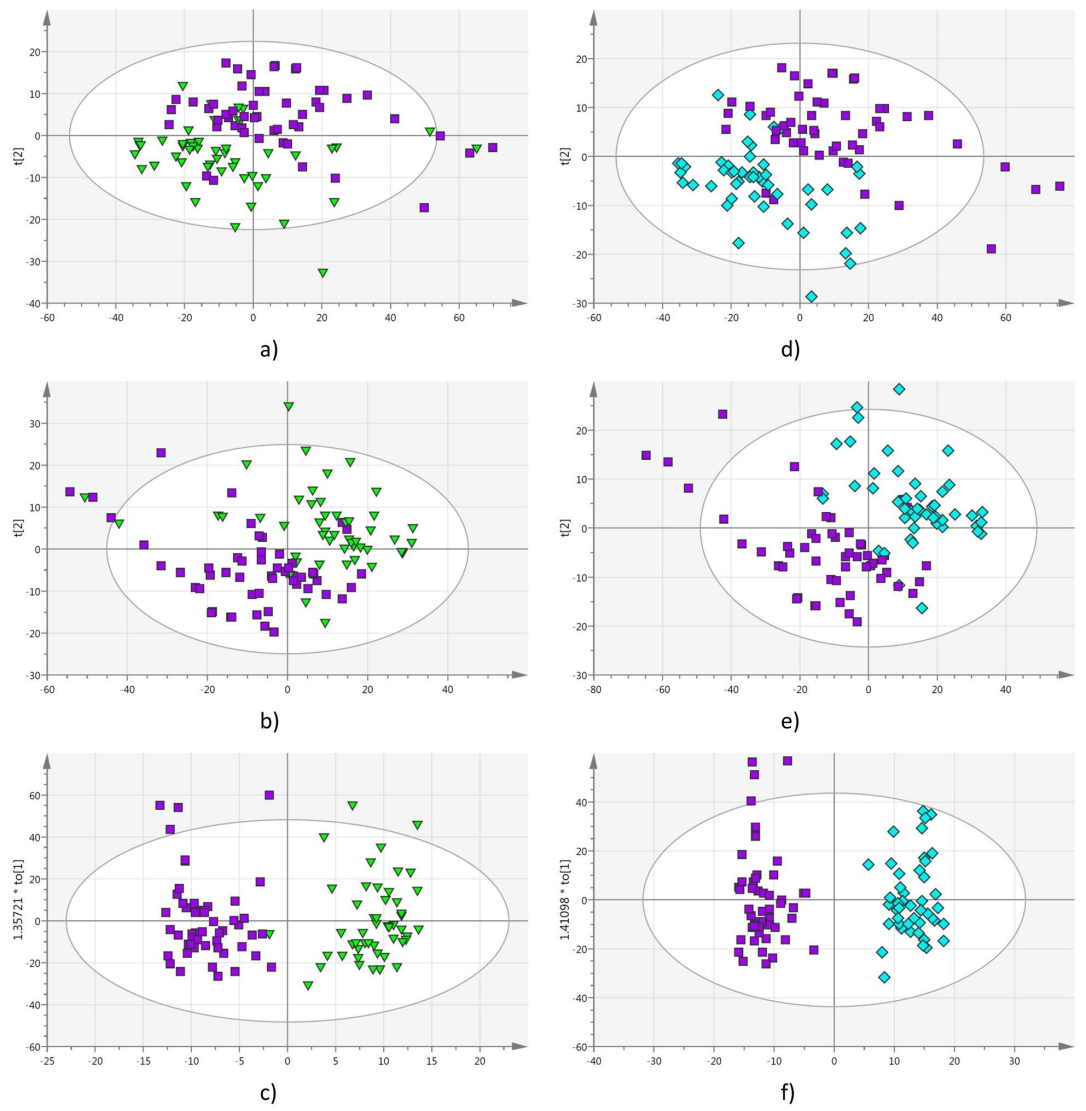
(**a**) PCA Scores plot; (**b**) PLS-DA Scores plot; (**c**) OPLS-DA Scores plot; analysis comparing UV–Vis spectra of pomegranate juices (squares) to those of 30% (inverted triangles) mixtures. (**d**) PCA Scores plot; (**e**) PLS-DA Scores plot; (**f**) OPLS-DA Scores plot; analysis comparing UV–Vis spectra of pomegranate juices (squares) to those of 40% (diamonds) mixtures.

**Figure 4:**
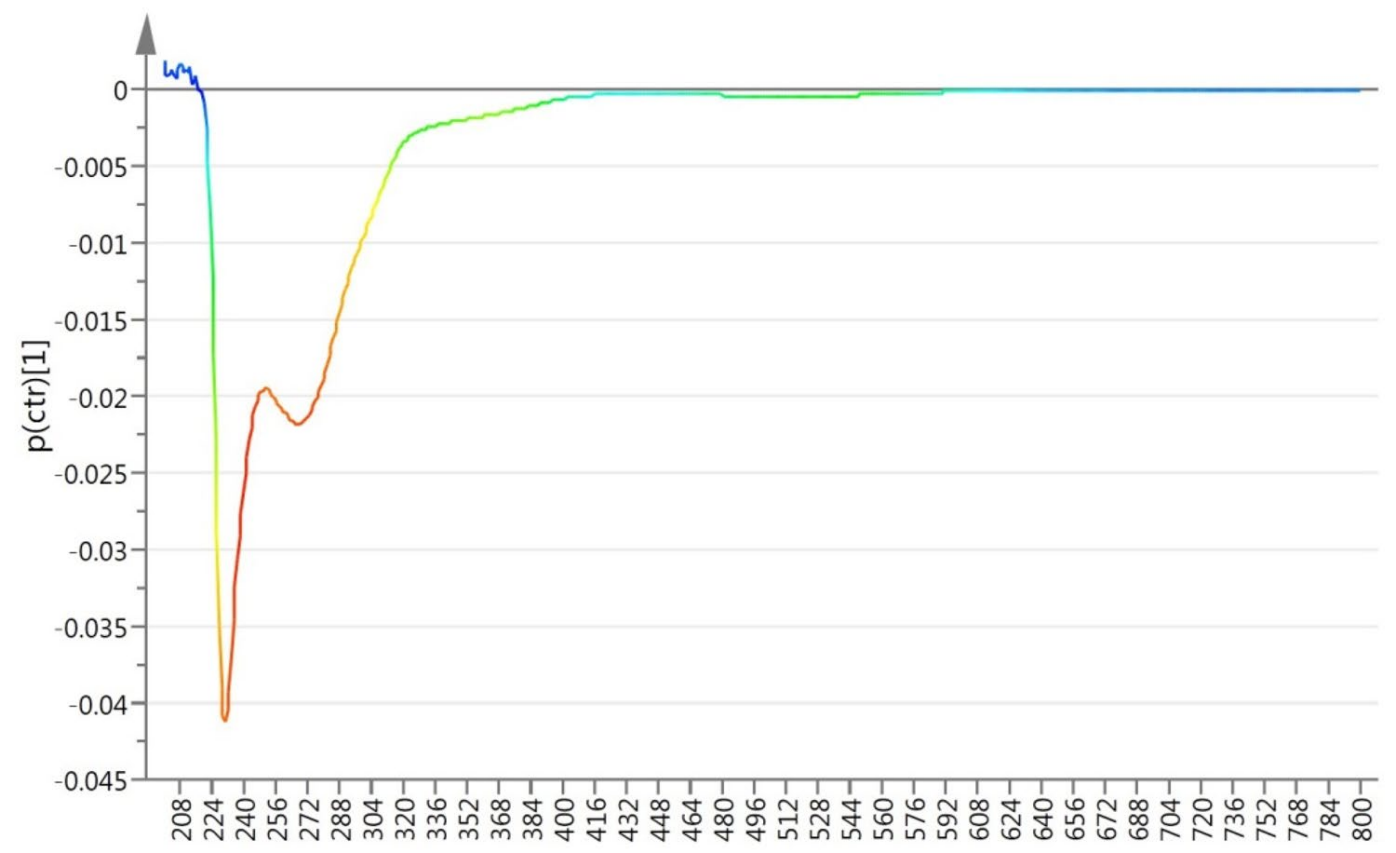
Coefficient plot derived from the OPLS-DA multivariate statistical analysis of UV–Vis spectra of pomegranate juices vs 30% v/v apple/pomegranate juice mixtures. The spectral variables responsible for the separation among the groups are represented in red. These are the regions of the spectra mostly affected by the adulteration.

In order to determine if pomegranate juice adulterated by apple juice can be detected by this method, the mixtures at 10%, 20%, 30% and 40% v/v apple/pomegranate juices were then analyzed. The multivariate statistical analysis was applied to two groups at the time, where the pure pomegranate juices spectroscopic data were analyzed with respect to each mixture. Figures 2 and 3 show the corresponding PCA, PLS-DA and OPLS-DA scores plots for the analysis of pomegranate juices (P) with respect to mixtures of respectively 10% (A10) (Fig. 2a–c), 20% (A20) (Fig. 2d–f), 30% (A30) (Fig. 3a–c) and 40% (A40) (Fig. 3d–f) v/v apple/pomegranate juices. The R^2^ (cum) and Q^2^ (cum) values for the overall analyses are summarized in Table 1. The analyses show that the cluster correlation [R^2^ (cum)] and predictability [Q^2^ (cum)] increase as the percentage of apple juice in the mixtures increases, i.e. the method works better greater the amount of the filler (apple juice). The coefficient plots indicated that the spectral variables of importance responsible for the separation belong to the 225–305 nm region (Fig. 4). The second set of experiments showed that supervised OPLS-DA analysis applied to UV–Vis spectra data was able to detect pomegranate juices adulterated by more than 20% v/v apple juices. While unsupervised PCA analysis alone proved to being unable to properly detect pomegranate juices adulterated with up to 40% v/v apple juice (Figs. 1S–10S).

**Table 1:** Goodness of fit of the data and predictability of the models.

Groups*	PLS-DA		OPLS-DA	
	R^2^ (cum)	Q^2^ (cum)	R^2^ (cum)	Q^2^ (cum)
P vs A10	0.277	0.193	0.300	0.222
P vs A20	0.513	0.446	0.846	0.748
P vs A30	0.910	0.769	0.888	0.832
P vs A40	0.962	0.914	0.948	0.922
P vs A	0.991	0.987	0.991	0.989

*Pomegranate juices (P), apple juices (A), 10% (A10), 20% (A20), 30% (A30), 40% (A40) v/v apple/pomegranate mixtures.

## Conclusions

Detection of adulteration in fresh fruit juices has become of broad interest to consumers, retailers and producers. The possibility to develop inexpensive methods to rapidly detect samples that have been adulterated would represent a major goal in this sector.

In this study UV–visible spectroscopy was combined with supervised and unsupervised multivariate statistical analysis to detect pomegranate juices adulterated with less expensive apple juice concentrate. The analyses show that OPLS-DA applied to UV–Vis spectra can only detected pomegranate fruit juices adulterated by more than 20% v/v apple juices, while unsupervised PCA is unable to properly detect pomegranate juices adulterated with up to 40% v/v apple juice.

## Supplementary Information

Supplementary Information.

## Data Availability

Data is available upon reasonable request.
