# Supplementary material for: Pomegranate fruit juice adulteration with apple juice: detection by UV–visible spectroscopy combined with multivariate statistical analysis
Source: Sci Rep. 2022 Mar 25;12:5151. doi: 10.1038/s41598-022-07979-7 (PMC8956635; doi:10.1038/s41598-022-07979-7)
Supplement: Supplementary file 1 — Supplementary Information. [file 41598_2022_7979_MOESM1_ESM.docx]

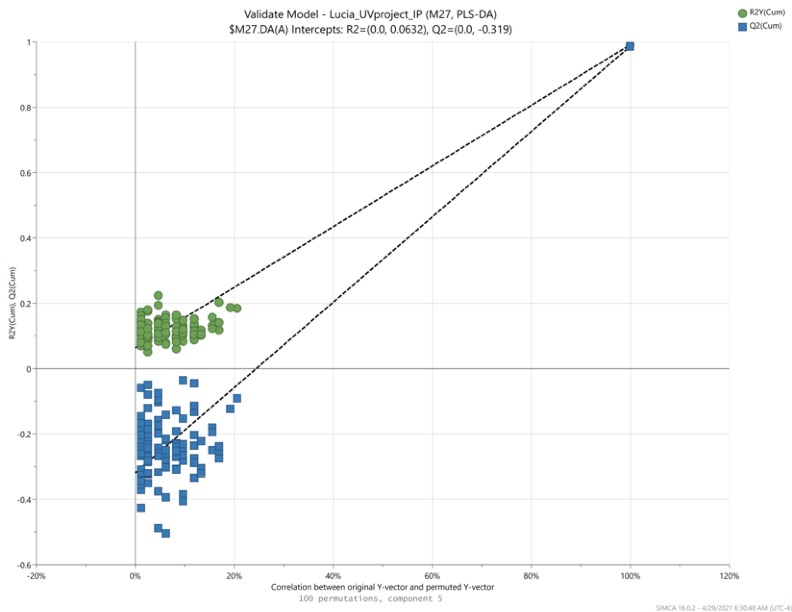


**Fig. 1S** Validation Plots; PLS-DA analysis comparing UV-VIS spectra of pomegranate juices to apple juices.


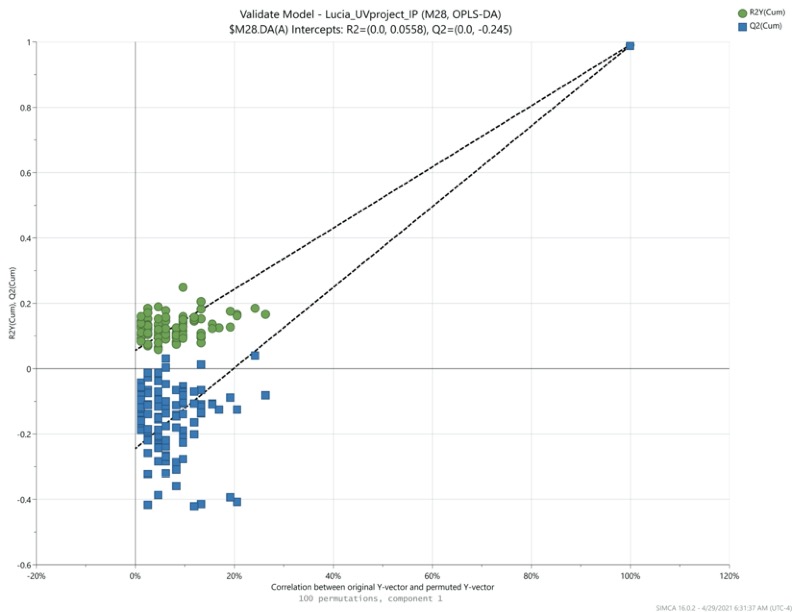


**Fig. 2S** Validation Plots; OPLS-DA analysis comparing UV-VIS spectra of pomegranate juices to to apple juices.


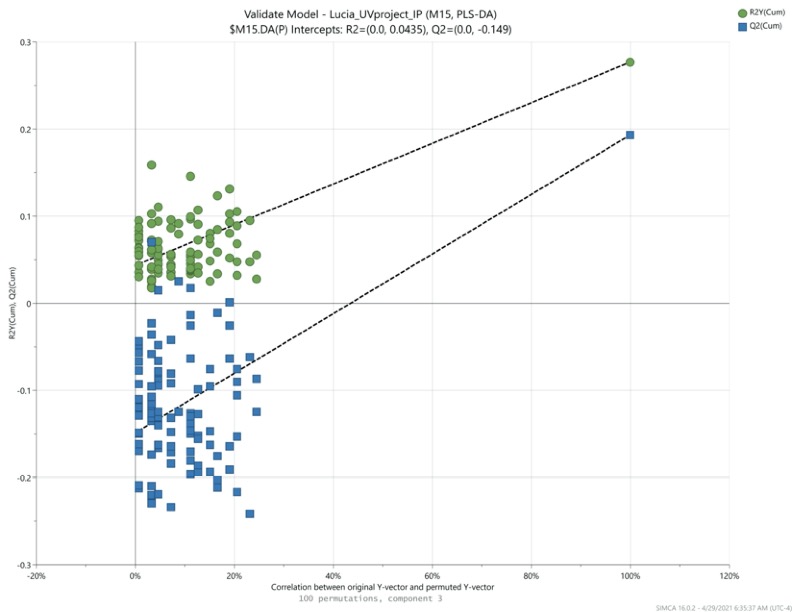


**Fig. 3S** Validation Plots; PLS-DA analysis comparing UV-VIS spectra of pomegranate juices to those of 10% mixtures.


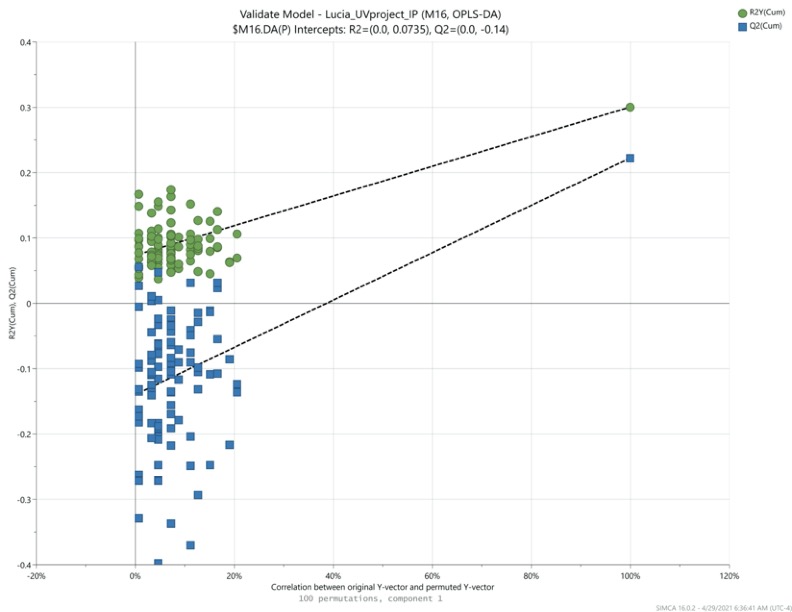


**Fig. 4S** Validation Plots; OPLS-DA analysis comparing UV-VIS spectra of pomegranate juices to those of 10% mixtures.


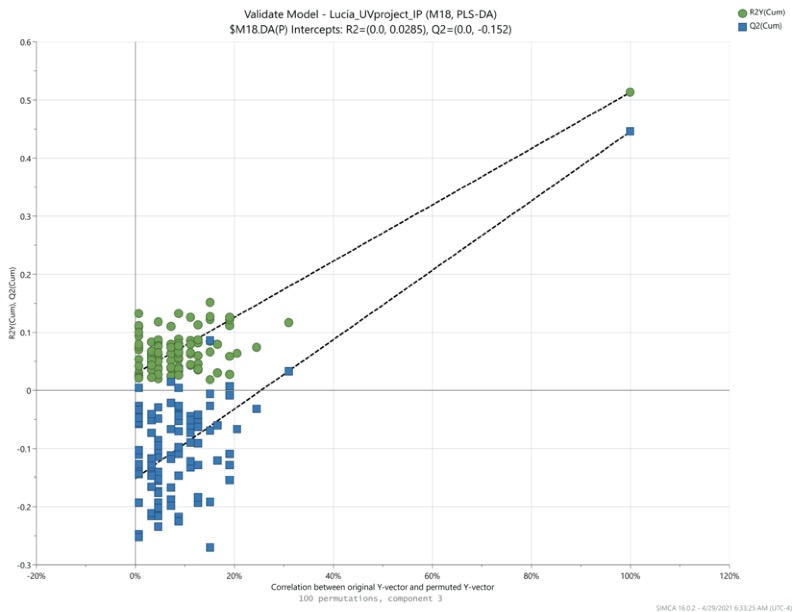


**Fig. 5S** Validation Plots; PLS-DA analysis comparing UV-VIS spectra of pomegranate juices to those of 20% mixtures.


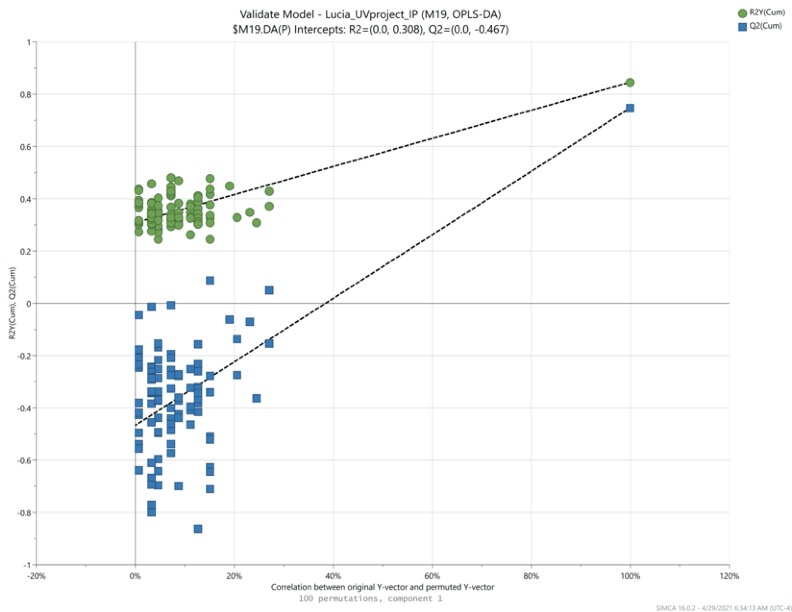


**Fig. 6S** Validation Plots; OPLS-DA analysis comparing UV-VIS spectra of pomegranate juices to those of 20% mixtures.


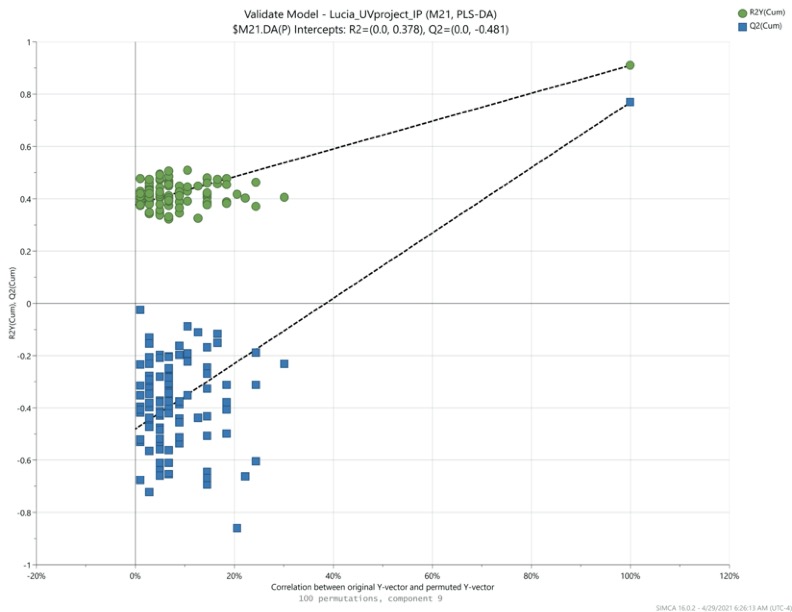


**Fig. 7S** Validation Plots; PLS-DA analysis comparing UV-VIS spectra of pomegranate juices to those of 30% mixtures.


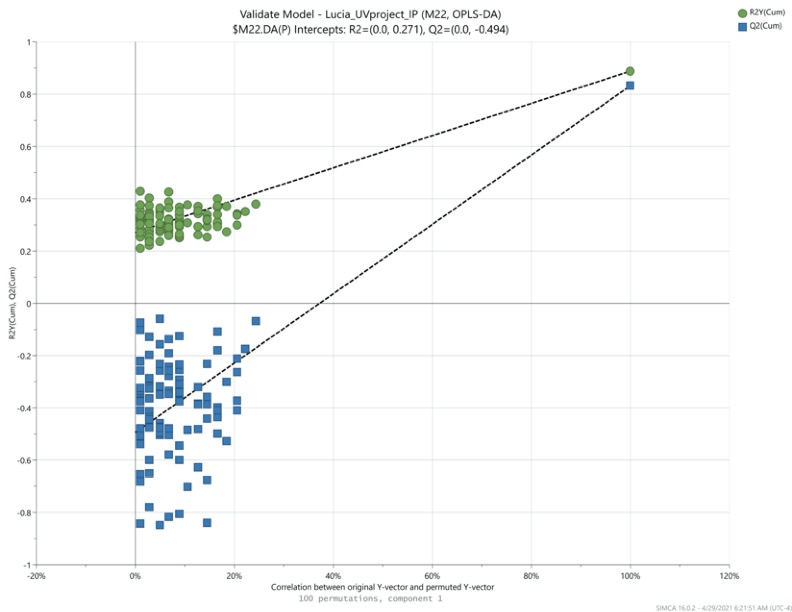


**Fig. 8S** Validation Plots; OPLS-DA analysis comparing UV-VIS spectra of pomegranate juices to those of 30% mixtures.


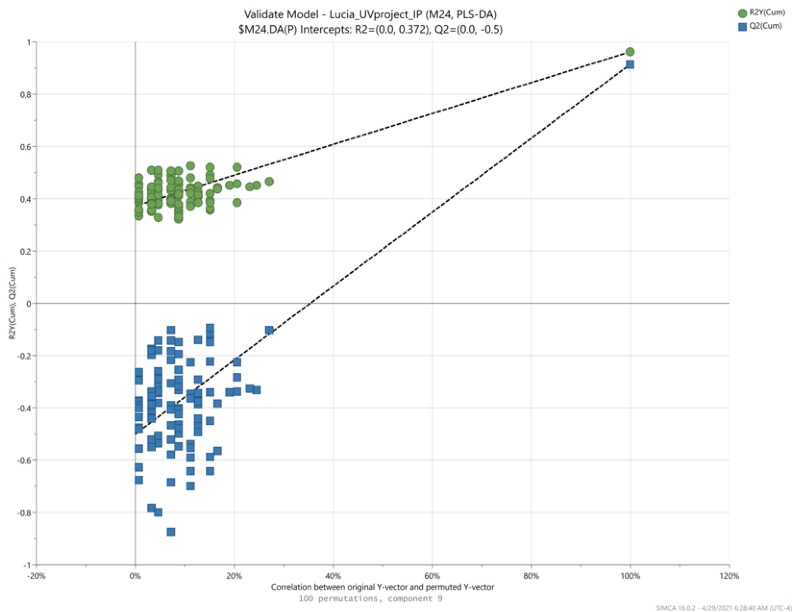


**Fig. 9S** Validation Plots; OPLS-DA analysis comparing UV-VIS spectra of pomegranate juices to those of 40% mixtures.


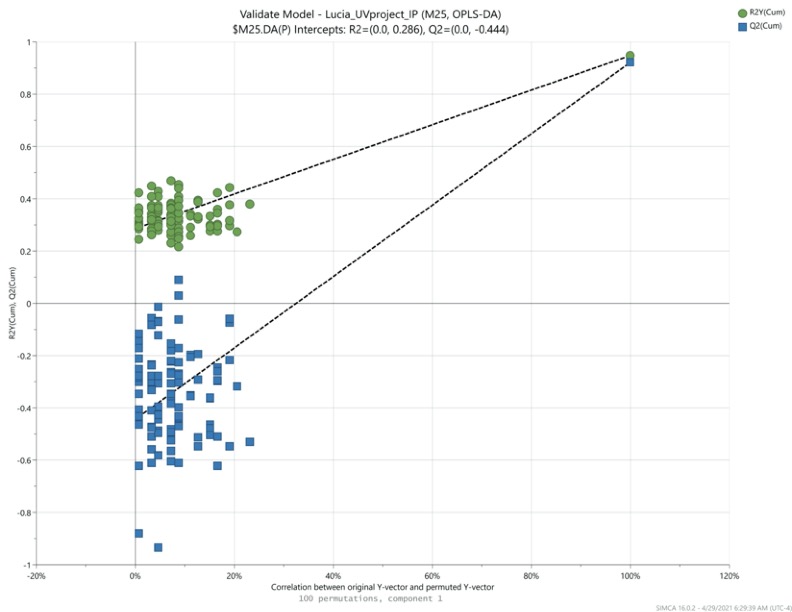


**Fig. 10S** Validation Plots; OPLS-DA analysis comparing UV-VIS spectra of pomegranate juices to those of 40% mixtures.

Receiver Operating Characteristics (ROC) values -- AUC, Area Under the Curve

%             P              A                                       R2X representation along the first/second component in PCA scores

10           0.903        0.272                                                                           76.2%/11.8%

20           0.963        0.419                                                                           75.1%/14.0%

30           0.967        0.577                                                                           76.4%/13.4%

40           0.953        0.765                                                                           76.5%/14.3%

1:1          0.860        1.000                                                                           89.3%/6.8%
